# Prognostic Performance of Bedside Lung Ultrasound Score (LUSS) and ROX Index in Hypoxemic Respiratory Failure Due to COVID-19

**DOI:** 10.3390/diagnostics13071361

**Published:** 2023-04-06

**Authors:** Alice Nova, Emanuele Rezoagli, Nilde Eronia, Annalisa Benini, Andrea Scognamiglio, Giuseppe Foti, Giacomo Bellani

**Affiliations:** 1 School of Medicine and Surgery, University of Milano-Bicocca, 20900 Monza, Italy; 2 Department of Emergency and Intensive Care, Terapia Intensiva e Semintensiva Adulti e Pediatrica, Fondazione IRCCS San Gerardo dei Tintori, 20900 Monza, Italy; 3 Centre for Medical Sciences-CISMed, University of Trento, Via S. Maria Maddalena 1, 38122 Trento, Italy; 4 Anesthesia and Intensive Care, Santa Chiara Regional Hospital, APSS (Azienda Provinciale per i Servizi Sanitari), 38123 Trento, Italy

**Keywords:** ROX, LUSS, ultrasound, noninvasive ventilation, intubation, mortality, survival

## Abstract

*Background:* Noninvasive ventilation, mainly helmet CPAP, was widely used during the COVID-19 pandemic, even outside of intensive care units. Both the ROX index and the LUS score (LUSS) have been proposed as tools to predict negative outcomes in patients with hypoxemia treated with noninvasive ventilation (NIV) outside of ICUs. We aim to evaluate whether the combination of LUSS with the ROX index improves the predictive performance of these indices in patients with hypoxemia due to COVID-19 pneumonia, treated with NIV outside of ICUs. *Methods:* This is a monocentric prospective observational study conducted at the university teaching hospital Fondazione IRCCS San Gerardo dei Tintori (Monza, Italy) from February to April 2021. LUSS and ROX were collected at the same time in noninvasively ventilated patients outside of the ICU. An LUS exam was performed by 3 emergency medicine attending physicians with at least 5 years’ experience in point-of-care ultrasonography using a 12-zone system. To evaluate the accuracy of the prognostic indices in predicting a composite outcome (endotracheal intubation and mortality), ROC curves were used. A logistic multivariable model was used to explore the predictors of the composite outcome of endotracheal intubation and in-hospital mortality. An unadjusted Kaplan–Meier analysis was used to explore the association with the composite outcome of survival without invasive mechanical ventilation at the 30-day follow-up by stratifying the 3 indices by their best cut-offs. *Results:* A total of 79 patients were included in the statistical analysis and stratified into 2 groups based on the presence of a negative outcome, which was reported in 24 patients out of 79 (30%). A great proportion of patients (66 patients—84%) were treated with helmet CPAP. All three indices (LUSS, ROX and LUSS/ROX) were independently associated with negative outcomes in the multivariable analyses. Although the comparison between the AUROC of LUSS or ROX versus LUSS/ROX did not reveal a statistically significant difference, we observed a trend toward a higher accuracy for predicting negative outcomes using the LUSS/ROX index as compared to using LUSS. With the Kaplan–Maier approach, all three indices stratified by the best cut-off reported a significant association with the outcome of 30-day survival without mechanical ventilation. *Conclusions:* A multimodal noninvasive approach that combines ultrasound (i.e., LUSS) and a bedside clinical evaluation (i.e., the ROX index) may help clinicians to predict outcomes and to identify patients who would benefit the most from invasive respiratory support.

## 1. Introduction

Coronavirus disease (COVID-19) rapidly spread worldwide and constituted a public health emergency. The clinical presentation is highly variable, with most people with the infection expressing mild-to-moderate symptoms and about 20% of patients developing severe respiratory failure [1]. Acute respiratory failure due to COVID-19 may cause profound hypoxemia, chest X-ray infiltrates and dyspnea with a high morbidity and mortality [2].

Noninvasive ventilation (NIV) was widely used during the COVID-19 pandemic, when an overwhelming number of patients needed respiratory assistance. The rapid shortage of intensive care unit (ICU) beds forced healthcare professionals to apply different types of noninvasive respiratory support, including helmet continuous positive airway pressure (CPAP), noninvasive pressure support ventilation (NPPV) and high-flow nasal oxygen (HFNO), not only in ICUs but also in emergency departments and regular wards [3,4].

The respiratory rate–oxygenation (ROX) index has been proposed as an easy tool to identify patients treated with noninvasive ventilation at high risk of worsening. The ROX index has been shown to predict treatment failure and the need for endotracheal intubation within 12 h from HFNO onset [5]. Recently, the role of the ROX index in identifying patients with COVID-19 with dyspnea at high evolutionary risk has been demonstrated [6].

Ultrasonography is a feasible bedside and radiation-free tool to monitor the clinical course of respiratory failure at any time. Lung ultrasound (LUS) is widely used as a diagnostic technique and as a guide in quick therapeutic decision making in critical care settings. It is an interesting tool in the evaluation of respiratory failure etiology [7] and has been recently demonstrated to be a useful monitoring device for the clinical history of ARDS [8,9,10].

During the COVID-19 pandemic, the clinical use of the LUS score (LUSS) increased. The LUS findings described in COVID-19 pneumonia include vertical pneumogenic artifacts (B-line patterns), subpleural consolidation and alterations of the pleural line, similar to those described in patients with non-COVID pneumonia [11,12,13]. In COVID-19 respiratory failure, LUS provides risk stratification, as it has been demonstrated to be a good predictor of death, ICU admission and endotracheal intubation [14,15,16]. In this context, LUS may help clinicians to predict the clinical course of patients in and out of ICUs.

The aim of the present study is to evaluate whether the effect of incorporating the LUS score (LUSS), a semiquantitative score that measures lung aeration loss caused by different pathological conditions [7,17], in the already known ROX index improves its predictive performance in patients with hypoxemic respiratory failure due to COVID-19 pneumonia, treated with NIV outside of ICUs. We defined the LUSS/ROX index as the ratio between the LUS score, performed using a 12-zone scoring system that includes an ultrasound of the lung parenchyma and pleural line patterns, and the ROX index.

## 2. Materials and Methods

This is a monocentric prospective observational study conducted at the university teaching hospital Fondazione IRCCS San Gerardo dei Tintori, Monza, Italy, which served as a referral center for COVID-19 pneumonia during the COVID-19 pandemic. Data were collected from February to April 2021. The study was conducted in accordance with the Declaration of Helsinki. Patients’ consent was waived due to the observational nature of the study and according to the Institutional Review Board approval. Data were collected as part of the STORM study (Spallanzani Institute approval number 84/2020; NCT04424992).

The inclusion criteria were as follows:

- Age ≥ 18 years;
- Confirmed COVID-19 pneumonia (positive test result using reverse-transcriptase polymerase chain reaction, RT-PCR, assay of a nasopharyngeal swab); Alert of the hospital Medical Emergency Team for clinical worsening: PaO_2_/FiO_2_ ratio < 300 mmHg despite oxygen therapy, respiratory rate > 22 breaths per minute and/or respiratory distress.

The exclusion criteria were hemodynamic instability requiring vasoactive drugs and comorbidities with a life expectancy < 12 months. All eligible patients were enrolled.

The following variables were collected: sex, age, body mass index (BMI), sequential organ failure assessment (SOFA) score, C-reactive protein (PCR) and applied oxygen support devices (i.e., HFNO, CPAP helmet and Venturi mask).

### 2.1. LUS Protocol

An LUS exam was performed within 4 days of hospital admission using a pre-defined step-by-step protocol as part of routine patient care management. A total of 3 emergency medicine attending physicians with at least 5 years’ experience in point-of-care ultrasonography collected the US parameters. The patient was preferably examined in the sitting position or, when this position could not be maintained, in the semirecumbent position. We performed LUS applying a six-zone method for each lung. We obtained 12 zones, if considering the whole thorax. We explored the anterior, antero-lateral and posterior areas of the thorax. A 12-4 Mhz linear-array transducer was used (Lumify L12-4, (c) Philips, Amsterdam, The Netherlands), adjusting the portable sonography machine in the lung preset to a reasonable depth.

We graded each patient by performing a 12-zone scoring system (lung ultrasound score, LUSS), including an ultrasound of the lung parenchyma and pleural line patterns, according to the current literature that describes the utility of LUS in predicting the clinical course of patients with COVID-19 [11,14,15,16,17].

- Lung parenchyma score: A score of 0 was assigned for no B-lines in a single intercostal space, a score of 1 was assigned for multiple spaced or isolated B-lines, a score of 2 was assigned for diffuse coalescent B-lines, and a score of 3 was assigned for lung consolidations.
- Pleural line score: A score of 0 was assigned for a normal, continuous, hyperechoic pleural line; a score of 1 was assigned for a discontinuous, irregular pleural line; and a score of 2 was assigned for a broken or a blurred pleural line.

The final LUSS was the sum of the points in all 12 regions, and it ranged from 0 to 60 [12]. If the maximum score was assigned to the lung (36 points) and to the pleura (24 points), an overall maximum score of 60 was obtained.

### 2.2. Clinical Data

At the time of the ultrasonography study, the respiratory rate, peripheral oxygen saturation (SpO_2_), oxygen device support used, fraction of inspired oxygen (FiO_2_) and positive end-expiratory pressure (PEEP), if applied, were obtained. Thus, an arterial gas sample was collected. The ROX index was calculated as the ratio between an oxygenation parameter, assessed using SpO_2_/FiO_2_, and the respiratory rate at the same time of the LUSS assessment.

We defined LUSS/ROX as the ratio between the LUS score and the ROX index, evaluated at the same time.

The patients were followed up at 30 days or until hospital discharge, and the need for intubation and status at hospital discharge (alive, transferred to another hospital or deceased) were recorded. The main study outcome was considered a composite outcome of the need for tracheal intubation within 30 days from the day of hospital admission and in-hospital mortality from any cause despite invasive or noninvasive ventilatory support.

### 2.3. Statistical Analyses

Normality was assessed using the Shapiro–Wilk test, and continuous data are expressed as mean ± SD or median [IQR], as appropriate. Categorical data are expressed as count (proportion). Comparisons between two groups of normally distributed variables were performed using the independent samples *t*-test, while non-normally distributed variables were compared using the Mann–Whitney U test. The correlations between the qualitative and quantitative variables were obtained using univariable and multivariable logic regression. The factors found to be associated with negative outcomes using univariable logic regression based on *p* < 0.1 and considered clinically relevant were included in the multivariable models. Thus, in the multivariable analysis, we included baseline patient characteristics (age and BMI); variables describing the respiratory illness severity (respiratory rate, PaO_2_/FiO_2_ and set PEEP); and ROX or, alternatively, LUSS or LUSS/ROX. Adjusted models were ranked by using the Akaike information criterion (AIC). AIC provides information on both the simplicity and goodness of fit of a model. As we compared multivariable models that included the same number of independent variables for the same set of patients, the best-fit model was represented by the lowest AIC. To evaluate the accuracy of the ROX index, LUSS and their ratio (LUSS/ROX) in predicting negative outcomes, receiver-operating characteristic (ROC) curves were used. We tested the difference between the area under the ROC areas (AUROCs) of ROX, LUSS and the LUS/ROX index by using the command “roccomp”. The best cut-off value to predict NIV failure or death was defined by Youden’s index calculation. A Kaplan–Meier survival analysis at the 30-day follow-up—available in all patients—was performed to determine the probability of survival without invasive mechanical ventilation in the overall population stratified by the best cut-offs of LUSS, the ROX index and the LUSS/ROX index to predict the composite outcome. Differences between the stratified populations was assessed using the log-rank test. The significance level was set to 5% (two-sided). Stata/MP version 17 (Copyright 1985–2021 StataCorp LLC, College Station, TX, USA) was used for the statistical analysis.

## 3. Results

### 3.1. Study Population

We enrolled 79 patients admitted to the Emergency Department of the university teaching hospital Fondazione IRCCS San Gerardo dei Tintori (Monza, Italy) from February to April 2021. All enrolled patients were included in the statistical analysis. All patients had COVID-19 pneumonia and were treated with noninvasive respiratory support outside of the ICU. We stratified the patients into 2 groups based on the presence of the composite outcome of endotracheal intubation and mortality, which was described in 24 patients out of 79 (30%).

Table 1 summarizes the main demographic variables, comorbidities and respiratory parameters of our cohort. We observed more immunosuppressed patients and a trend of a higher BMI in the negative-outcome group. Organ dysfunction severity expressed by the SOFA score was homogeneous between the two groups. We did not observe a difference in laboratory biomarkers at baseline between the study groups.

### 3.2. Ventilatory Variables and Outcomes

A great proportion of patients (66 patients—84%) were treated with helmet CPAP. In all of them, the in-flow gas was delivered through a free-flow system and a PEEP valve with a median set PEEP of 8 cmH_2_O [IQR, 5–10]. A Venturi mask was used in 10 (13%) patients, and 3 patients (4%) were treated with HFNO. The median FiO_2_ was 0.6 [IQR, 0.5–0.8]. Mild hypoxemia was observed in the overall population (PaO_2_/FiO_2_ of 245 ± 101 mmHg). The patients who failed NIV or died had a significantly lower PaO_2_/FiO_2_ ratio, a higher respiratory rate and PEEP, and a lower ROX index. LUSS and the LUSS/ROX index were significantly higher in the patients with a negative outcome.

### 3.3. Multivariable Analyses

The multivariable analyses (Table 2) showed that all three indices (ROX, LUSS and LUSS/ROX) were independently associated with negative outcomes. When comparing the models, the Akaike information criterion (AIC) favored the model exploring LUSS/ROX over the model assessing LUSS alone.

### 3.4. Prognostic Performance of the 3 Indices

**Figure 1:**
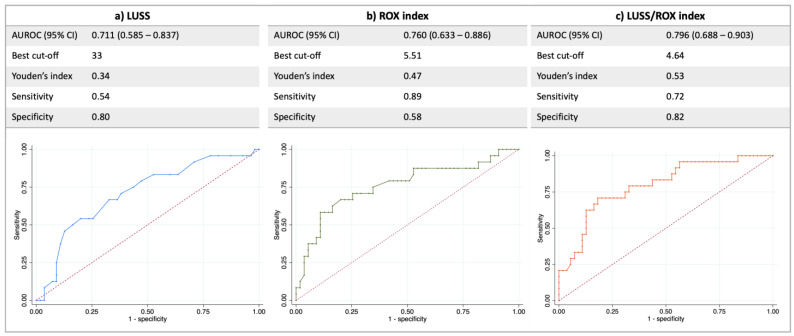
AUROC, best cut-off, sensitivity and specificity of LUSS (**a**), ROX (**b**) and LUSS/ROX (**c**) in predicting the composite outcome of endotracheal intubation and mortality. *Definition of abbreviations:* AUROC: area under the receiver-operating characteristic curve; LUSS: lung ultrasound score; ROX: respiratory rate–oxygenation.

Figure 1 describes the optimal cut-off point (obtained using the highest Youden’s index), the sensitivity, the specificity and the area under the receiver-operating characteristic curve (AUROC) for the LUSS, the ROX index and the LUSS/ROX index. Although the comparison between the AUC of LUSS/ROX versus the ROX index and that versus LUSS did not reveal a statistically significant difference (*p* = 0.436 and *p* = 0.186, respectively), we observed a trend toward a higher accuracy for predicting a negative outcome using the LUSS/ROX index as compared with LUSS (Figure 1). A comparison between the AUCs of the single indices of LUSS versus the ROX index was not significant (*p* = 0.621).

### 3.5. Unadjusted Survival Analysis at 30-Day Follow-Up

**Figure 2:**
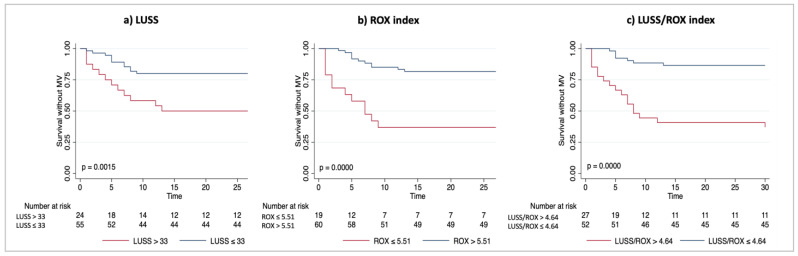
Unadjusted Kaplan–Meier plots showing the probability of survival without invasive mechanical ventilation according to LUSS (**a**), ROX index (**b**) and LUSS/ROX (**c**) stratified by their best cut-offs. *Definition of abbreviations:* MV: mechanical ventilation; LUSS: lung ultrasound score; ROX: respiratory rate–oxygenation. Differences between the stratified populations were assessed using log-rank test.

The Kaplan–Meier plots showed the probability of survival without invasive mechanical ventilation according to the LUSS, ROX and LUSS/ROX best cut-off groups (Figure 2). A statistically significant difference in survival without invasive mechanical ventilation was observed by stratifying the population using all three indices. We observed a higher probability of survival without mechanical ventilation over the 30-day follow-up by using the combined LUSS/ROX best cut-off (*p* = 0.0000 by log-rank test) (Figure 2c) than by using the best cut-off of LUSS (Figure 2a).

## 4. Discussion

In this monocentric prospective observational study conducted in noninvasively ventilated patients outside of the ICU, we observed the following:

− All three indices (ROX, LUSS and LUSS/ROX) were independently associated with a negative outcome in the univariable analysis, and this association was confirmed in the multivariable analyses.
− Although the comparison between the AUC of LUSS or ROX versus LUSS/ROX did not reveal a statistically significant difference, we observed a trend toward a better outcome prediction using the LUSS/ROX index as compared to using LUSS; furthermore, the AUROC of the combined LUSS/ROX index improved the low sensitivity of LUSS and the low specificity of the ROX index.
− Using the Kaplan–Meier approach, a statistically significant difference in survival without invasive mechanical ventilation was observed by stratifying the population using all three indices. We observed a higher probability of survival without mechanical ventilation over the 30-day follow-up by using the combined LUSS/ROX best cut-off (*p* = 0.0000 by log-rank test) (Figure 2c) when compared with LUSS best cut-off (*p* = 0.0015 by log-rank test) (Figure 2a).

Because of the overwhelming number of patients requiring respiratory support during the COVID-19 pandemic, a great proportion of hospitalized patients were treated outside of ICUs using noninvasive ventilatory support [3]. The helmet CPAP was applied in 83% of our patients, reflecting the common use of the device by healthcare professionals, the efficacy of PEEP in improving gas exchange and the advantage of reducing the risk of environmental contamination, in line with the literature [3]. In these patients, strict clinical monitoring is essential to predict treatment failure, to avoid self-inflicted lung injury and to not further defer intubation when required.

In this context, a lung ultrasound (LUS), well known for its feasibility at the bedside, noninvasiveness and radiation sparing effect, has been proposed to evaluate the respiratory failure severity [12,13]. LUS has been demonstrated to predict negative outcomes (i.e., endotracheal intubation and mortality) in patients with COVID-19 at the ED [14,15,18,19] and at ICU [15] admission. In our study, we evaluated the performance of a 12-zone scoring system (LUSS) in predicting respiratory deterioration in patients treated with noninvasive ventilation outside of ICUs and followed up by the Medical Emergency Team. We identified 33 as the threshold above which the risk of an adverse outcome increases, and LUSS was shown to predict negative outcomes with an AUC of 0.71 and a sensitivity of 80% (Figure 1a). Our cut-off is higher than the one described in the previous literature using the same scoring system (33 in our study vs. 12 described by L. Ji et al.) [18]. This finding may be related to a more severe respiratory disease in our population or to the presence of pulmonary co-existing conditions, considering that comorbidities potentially influencing image acquisition (i.e., heart failure, non-COVID 19 interstitial pneumonia and pleural effusion) were excluded by Li J. et al. in the design of their study [18]. LUSS showed a good level of accuracy in the stratification risk of patients treated with NIV in our cohort, constituted mainly of patients undergoing helmet CPAP, confirming the recent literature [14,15].

Another useful tool for the prognostication of noninvasively ventilated patients is the ROX index. In recent studies, it has been observed that the ROX index may help clinicians to identify patients at high risk of NIV failure during treatment with both HFNO [18] and helmet CPAP [6,19]. The threshold below which the risk of treatment failure is high and the ROX index is accurate for predicting negative outcomes in our study is similar to the one described in patients with hypoxemia and COVID-19 by Colaianni-Alfonso et al. after 12 h of CPAP treatment [6], as shown in Figure 1b. However, our cut-off is higher than the one proposed for non-COVID-19 patients receiving HFNC (3.85) [5]. This result may be related to the effect of positive airway pressure on arterial oxygenation and, thus, on peripheric saturation, which may not always be predictive of treatment success, especially in patients with COVID-19 [20].

We explored whether combining the LUS score with the ROX index, using the ratio between the two values obtained at the same time, improve the predictive performance of the two indices when used alone in patients undergoing NIV. We observed an independent association with negative outcomes for all three indices (Table 2), and the Akaike information criterion (AIC) favored the model including LUSS/ROX over the model including LUSS. Although the comparison between the three AUCs did not reach statistical significance, probably due to the small sample size, a trend toward a higher accuracy for predicting negative outcomes using LUSS/ROX was shown with a higher gain in the prognostic performance of LUSS/ROX when compared with the LUS score. The Kaplan–Meier analysis showed that using the best cut-off of the combined LUSS/ROX index could identify subgroups of patients significantly associated with the outcome of survival without mechanical ventilation (Figure 2c).

Considering that the same PEEP level may generate different effects in different patients (the maintenance of recruitment vs. overdistention) [21] and that the real pressure within the CPAP helmet could be higher than the set PEEP level because of HEPA and HMEF filters [22], incorporating the set PEEP in the ROX index does not increase its capacity to discriminate between patients who would succeed with NIV and those who would fail with it, as recently demonstrated [23]. During NIV, when invasive monitoring (i.e., tidal volume and transpulmonary pressure) is lacking, the ultrasonography gives the clinician a chance to evaluate the effect of a therapeutic maneuver to directly assess the change in lung aeration, as already described in critically ill patients with ARDS [24]. Furthermore, ultrasonography also allows one to unveil the cause of sudden or progressive clinical worsening. Thus, it is reasonable to think that combining a straight clinical evaluation, provided by the ROX index, with a simple ultrasound scoring system (LUSS) may result in a gain in risk stratification and outcome prediction.

Although our study describes the novelty of the application of LUSS and LUSS/ROX during the follow-up of noninvasively ventilated patients outside of ICUs, it must be acknowledged that our research may have some limitations. First, it was a single-center study with a relatively limited sample size, which included only patients with COVID-19 in a limited-resource setting. Furthermore, repeated measures of ROX, LUSS and LUSS/ROX over time were not available, so we could not investigate whether the prognostic performances of these indices change over time. Third, although the LUS exam was performed by an expert clinician using a standardized procedure, it remains an operator-dependent method. In our study, only one clinician performed the LUS exam for each patient; thus, we could not test inter-observer agreement.

## 5. Conclusions

In conclusion, these preliminary findings suggest that a multimodal noninvasive approach, including a lung ultrasound and a bedside clinical evaluation with the combined LUSS/ROX index, may help clinicians to predict a higher risk of adverse outcomes. Furthermore, this would allow one to identify patients who would benefit from NIV and to not further defer invasive support, when required, in a COVID-19 population mainly ventilated with helmet CPAP.

## Figures and Tables

**Table 1:** Main demographic variables, comorbidities and respiratory parameters of enrolled patients.

	Overall Population *n* = 79	Negative Outcome *n* = 24 (30%)	Positive Outcome *n* = 55 (70%)	*p*-Value
*Demographics*				
Age, years	61 [52–71]	68 [59–75]	60 [50–70]	0.067
Sex, female, n	28 (35%)	9 (38%)	19 (35%)	0.801
BMI, Kg/m^2^	28 [26–33]	30 [27–34]	27 [25–32]	0.052
*Comorbidities*				
COPD	11 (14%)	3 (13%)	8 (15%)	0.809
Diabetes	7 (9%)	1 (4%)	6 (11%)	0.332
Chronic renal failure	1 (1%)	0 (0%)	1 (2%)	0.506
Hypertension	31 (39%)	11 (46%)	20 (36%)	0.428
Ischemic cardiomyopathy	3 (4%)	2 (8%)	1 (2%)	0.164
Neoplasia	2 (2%)	1 (4%)	1 (2%)	0.541
Immunosuppression	2 (2%)	2 (8%)	0 (0%)	0.030
*Ventilatory setting*				
Venturi mask	10 (13%)	1 (4%)	9 (16%)	0.143
HFNO	3 (4%)	0 (0%)	3 (5%)	
CPAP	66 (84%)	23 (96%)	43 (78%)	
PEEP, cmH_2_O	8 [5–10]	10 [8–10]	8 [5–10]	0.014
FiO_2_	60 [50–80]	75 [65–100]	50 [50–70]	<0.001
*Clinical illness severity*				
SOFA baseline	2 [2–2]	2 [2–2]	2 [1–2]	0.299
PCR (mg/L)	6.70 [3.40–12.27]	5.00 [3.37–12.90]	7.36 [3.87–11.78]	0.534
Lymphocytes (cells/mL)	853 (±316)	760 (±72)	889 (±41)	0.112
D-dimer (μg/mL)	345 [208–540]	386 [260–536]	334 [208–540]	0.431
PaO_2_, mmHg	131 [89–210]	118 [87–220]	134 [92–210]	0.561
PaCO_2_, mmHg	36 [33–40]	37 [33–41]	36 [33–40]	0.685
SpO_2_, %	99 [97–99]	98 [97–99]	99 [98–99]	0.326
PaO_2_/FiO_2_	245 (±101)	197 (±93)	266 (±98)	0.005
RR, breaths per minute	24 [20–28]	26 [22–31]	24 [20–26]	0.028
*NIV failure indices*				
ROX index	7.07 [5.51–9.80]	5.14 [3.43–7.01]	7.55 [6.25–10.00]	<0.001
LUSS	27 (±9)	31 (±8)	25 (±9)	<0.001
LUSS/ROX	3.70 [2.49–5.73]	6.02 [3.94–8.76]	3.43 [2.10–4.29]	<0.001

*Definition of abbreviation:* Continuous data are expressed as mean ± SD or median [IQR], as appropriate; categorical variables are reported as count (proportion) (n, %). BMI: body mass index; COPD: chronic obstructive pulmonary disease; CRP: C-reactive protein; FiO_2_: inspiratory fraction of oxygen; HFNO: high-flow nasal oxygen; LUSS: lung ultrasound score; PaCO_2_: arterial partial pressure of carbon dioxide; PaO_2_: arterial partial pressure of oxygen; PEEP: positive end-expiratory pressure; RR: respiratory rate; ROX: respiratory rate–oxygenation; SOFA: sequential organ failure assessment; SpO_2_: peripheral oxygen saturation. All continuous variables have non-normal distribution, but PaO_2_/FiO_2_ ratio, LUSS and lymphocytes are normally distributed. Unpaired t-test was used to estimate differences in PaO_2_/FiO_2_ ratio, LUSS and lymphocytes between the groups; Mann–Whitney test was used for all other continuous variables.

**Table 2:** Multivariable analysis of factors, including ROX index, independently associated with probability of negative outcome.

Variable	OR (95% CI)	*p*-Value	OR (95% CI)	*p*-Value	OR (95% CI)	*p*-Value
BMI	1.077 (0.94–1.23)	0.277	1.07 (0.95–1.22)	0.260	1.08 (0.94–1.23)	0.280
Age	1.013 (0.96–1.06)	0.604	1.01 (0.97–1.06)	0.248	1.01 (0.97–1.06)	0.591
PEEP	1.289 (1.03–1.62)	0.029	1.22 (0.97–1.53)	0.093	1.23 (0.98–1.54)	0.073
PaO_2_/FiO_2_	0.992 (0.99–1.00)	0.020	0.99 (0.99–1.00)	0.069	0.99 (0.99–1.00)	0.064
RR	0.974 (0.83–1.14)	0.749	1.13 (1.00–1.27)	0.047	1.02 (0.89–1.17)	0.775
ROX	0.706 (0.51–0.98)	0.039				
LUSS			1.08 (1.00–1.17)	0.045		
LUSS/ROX					1.35 (1.04–1.76)	0.023
AIC	84		85		84	

*Definition of abbreviations:* AIC: Akaike information criterion; BMI: body mass index; FiO_2_: inspiratory fraction of oxygen; LUSS: lung ultrasound score; PaO_2_: arterial partial pressure of oxygen; PEEP: positive end-expiratory pressure; ROX: respiratory rate–oxygenation; RR: respiratory rate.

## Data Availability

Data are available from the corresponding author upon reasonable request.
